# Supplementary material for: Common patterns of gene regulation associated with Cesarean section and the development of islet autoimmunity – indications of immune cell activation
Source: Sci Rep. 2019 Apr 18;9:6250. doi: 10.1038/s41598-019-42750-5 (PMC6472354; doi:10.1038/s41598-019-42750-5)

Supplementary Information

Common patterns of gene regulation associated with Cesarean section and the development of islet autoimmunity – indications of immune cell activation

M. Laimighofer [1,2], R. Lickert [3], R. Fuerst [3], F. J. Theis [1,2], C. Winkler [3], E. Bonifacio [4,5], A.-G. Ziegler [3], and J. Krumsiek [1,6,7,#]

[1] Institute of Computational Biology, Helmholtz Zentrum München, Neuherberg, Germany

[2] Department of Mathematics, Technische Universität München, Garching, Germany

[3] Institute of Diabetes Research, Helmholtz Zentrum München, and Forschergruppe Diabetes, Klinikum rechts der Isar, Technische Universität München, Germany

[4] DFG Center for Regenerative Therapies Dresden, Faculty of Medicine, Technische Universität Dresden, Dresden, Germany

[5] Paul Langerhans Institute Dresden, German Center for Diabetes Research (DZD), Technische Universität Dresden, Dresden, Germany

[6] German Center for Diabetes Research (DZD), Neuherberg, Germany

[7] Institute for Computational Biomedicine, Englander Institute for Precision Medicine, Department of Physiology and Biophysics, Weill Cornell Medicine, New York, USA

[#] corresponding author, jan.krumsiek@helmholtz-muenchen.de

<u>Supplementary Figure S1.</u> Detailed information on longitudinal sampling and on Cesarean section in the dataset.
A: Sample distribution per child. Markers indicate PBMC transcriptomics samples from children at that timepoint (blue = born by 'Cesarean section', grey = 'vaginal delivery'). **B:** Zoom into A for the first year of life. **C:** PBMC transcriptomics samples per child in the first year of life.

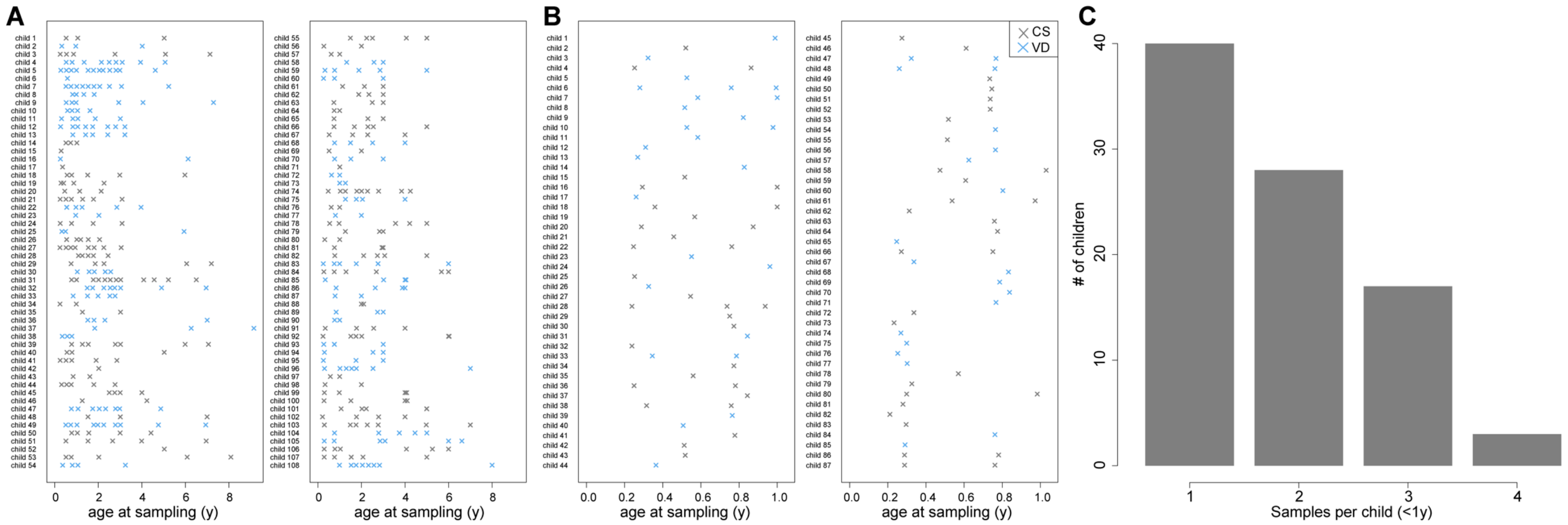

Supplementary Figure S2. Results of analysis for samples up to 6 months before islet autoimmunity compared to age matched islet autoantibody negatives children. Results of paired analysis of samples before and after seroconversion.

A: Histogram of p-values from samples up to 6 month before seroconversion vs. age-matched controls. Analysis corrected for age. **B:** Volcano plot of effect sizes and p-values. Dashed line indicates the multiple testing threshold (FDR = 0.05). **C**: Histogram of p-values from samples of children up to 6 months before seroconversion vs. samples up to 6 months after seroconversion in the same children (paired t-tests). **D:** Volcano plot of effect sizes and p-values of paired analysis. Dashed line indicates the multiple testing threshold (FDR = 0.05).

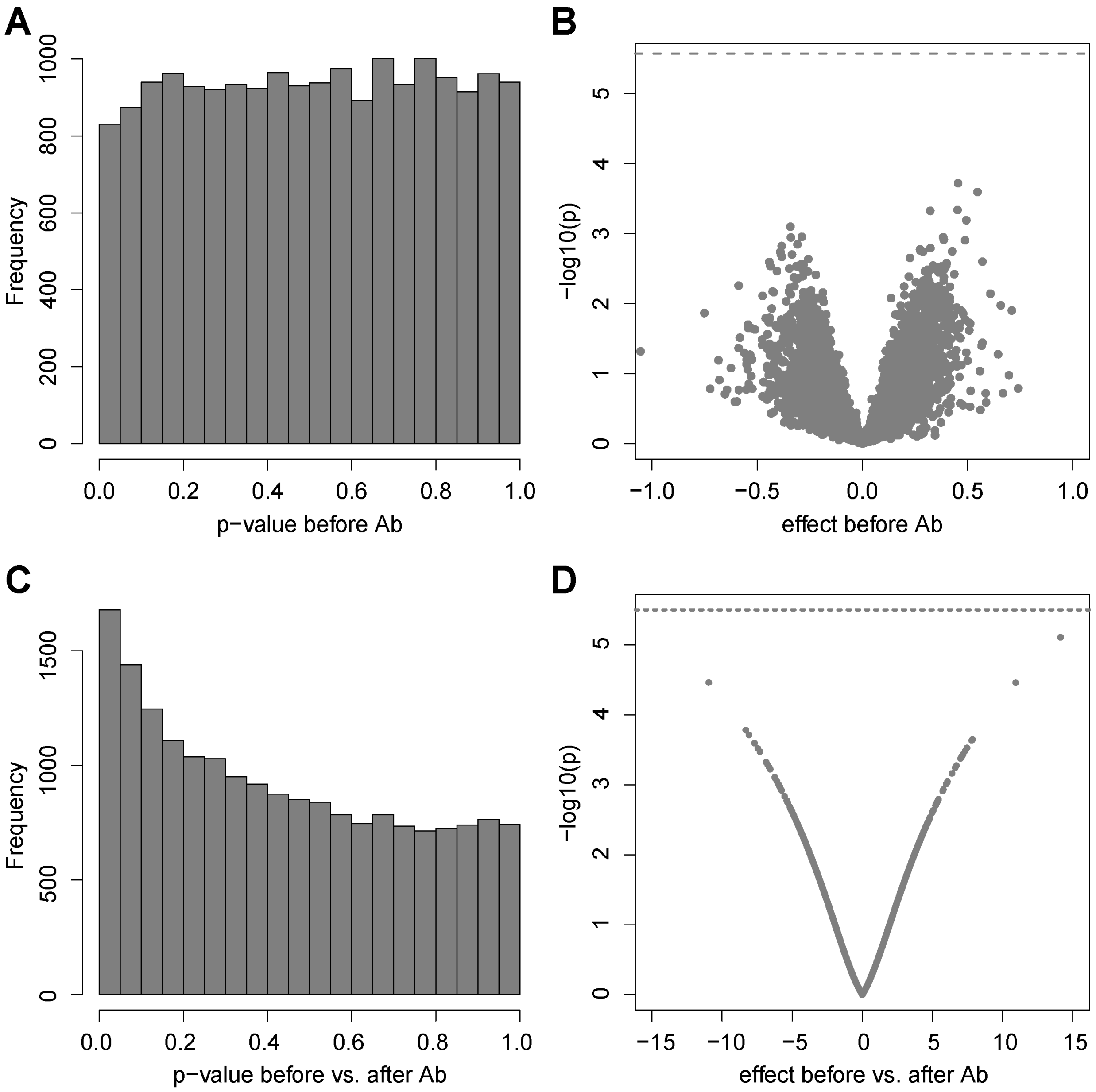

Supplementary Figure S3

Pentose phosphate pathway correlations between Cesarean section and islet autoantibody status.

Pentose phosphate pathway with two-sided node coloring according to directed p-values (-log10(p)*sign(t-statistic))

left-side: Cesarean section vs. vaginal delivery, right-side: islet autoimmunity status

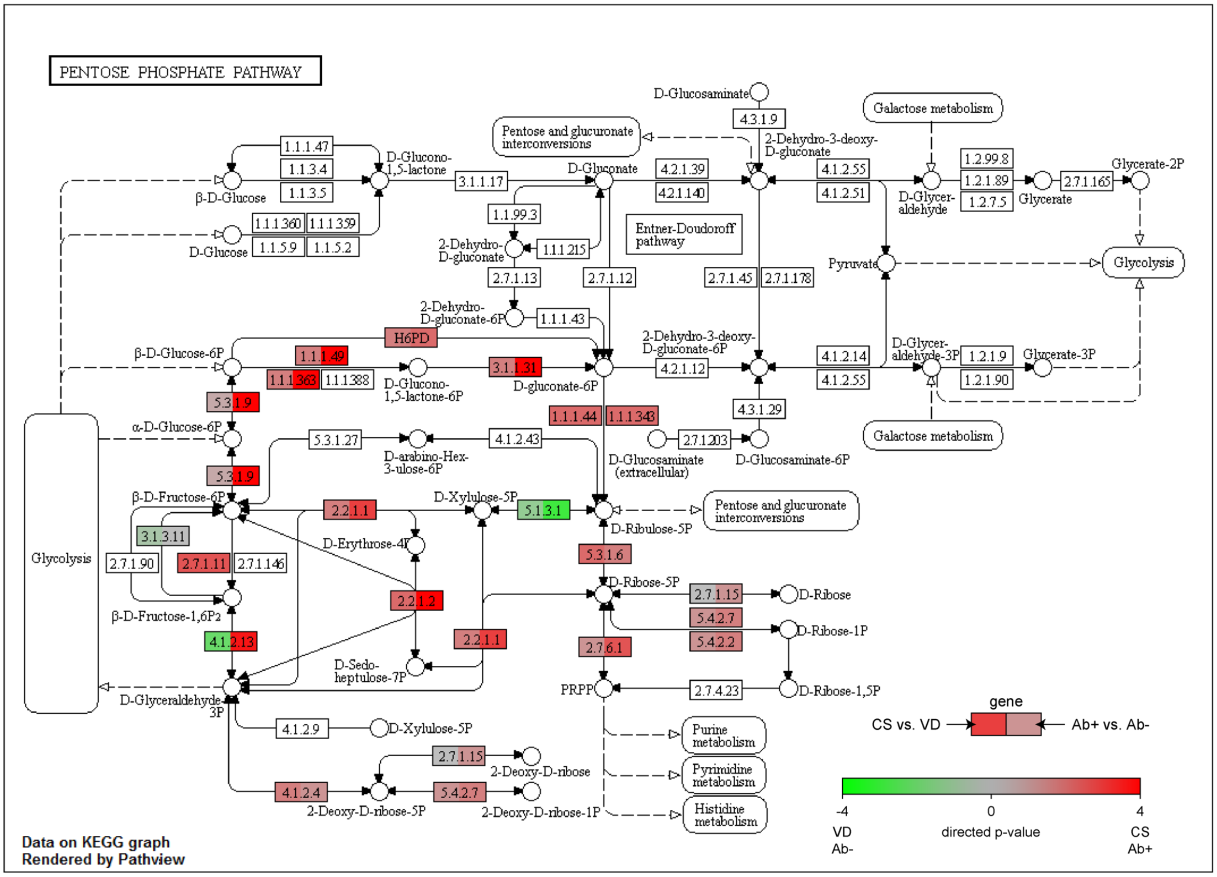

Supplementary Figure S4

Type of delivery (Cesarean section and vaginal delivery) vs. islet autoantibody status (Ab+ and Ab-). P-values are derived using a Fisher exact test.

N=109 (all children), p = 0.35	Autantibody negative	Autantibody positive
Vaginal delivery	51	11
Cesarean section	34	12

N=87 (children in set <1y), p = 0.32	Autantibody negative	Autantibody positive
Vaginal delivery	39	9
Cesarean section	34	11

N=89 (children in Ab status set), p = 0.25	Autantibody negative	Autantibody positive
Vaginal delivery	45	6
Cesarean section	29	8

Supplementary Figure S5

Permutation-based correlation analysis for maternal diabetes, gender and multiple first-degree relative. Histograms of correlation of effects between permuted class labels. Cesarean was correlated with **A:** maternal diabetes (MD); **B:** MD pathways; **C:** gender; **D:** gender pathways; **E:** multiple first-degree relatives (multiple FDR); **F:** multiple FDR pathways.

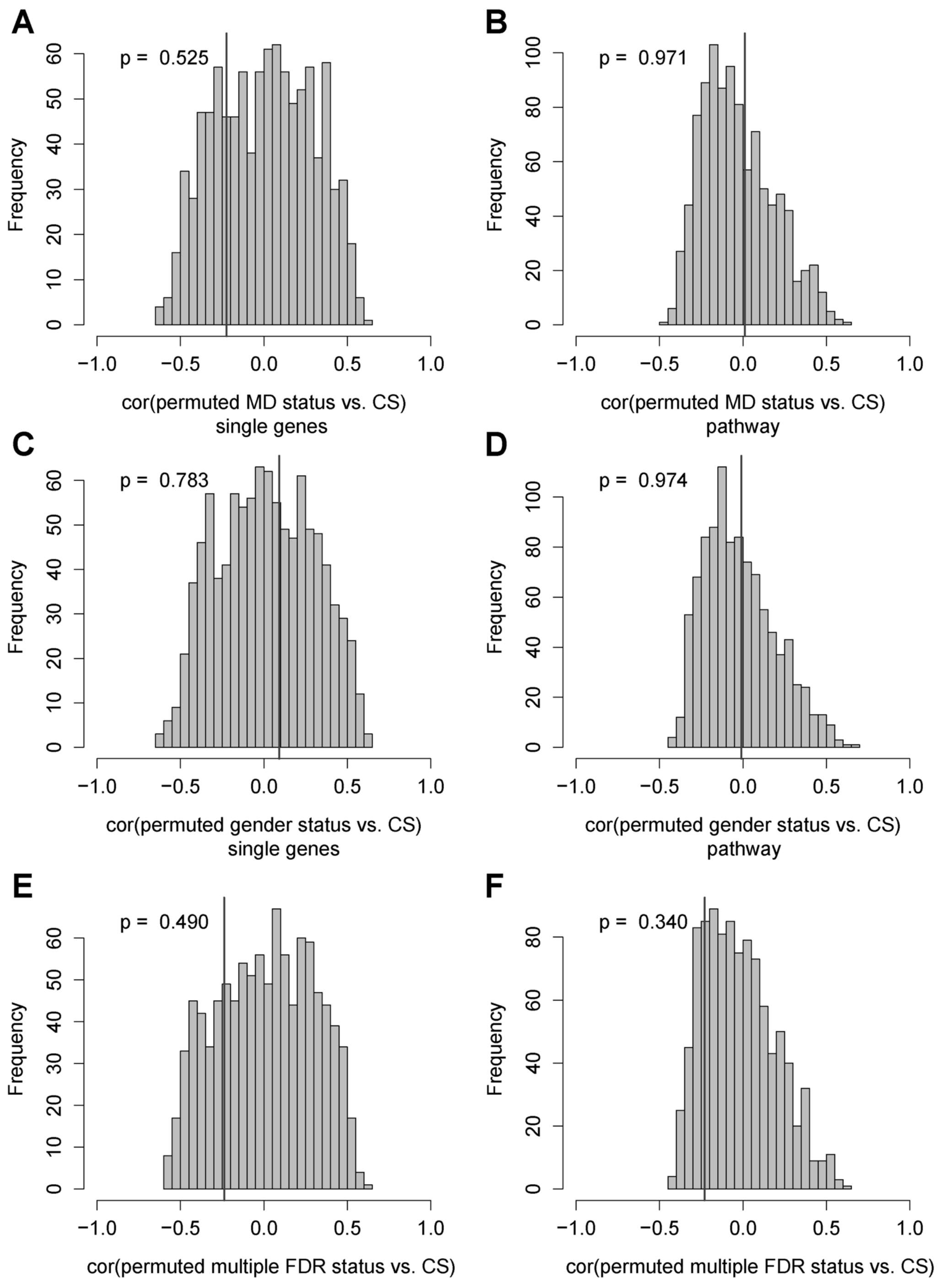

Supplementary Note S1. Single gene and pathway results of transcriptomics dataset GEO-22886 of activated immune cells.

In order to test the hypothesis of activation in Cesarean section and islet autoantibody positive children, we analyzed transcriptomics data of two different data sources: (1) Gene expression of primary human CD4+ T-cells, GEO-33272, see main manuscripts. (2) Gene expression profiling on various resting and activated human immune cells, GEO-22886.

In the following, we describe the results of the second dataset, where we analyzed three immune cell types, CD4+ T-cells, monocytes, natural killer cells, and the mixture of the three.

Results from GEO-22886

We restricted the analysis on a subset of cells: (1) resting CD4+ cells vs. activated Th1 and Th2 cells, (2) resting natural killer cells vs. IL2 activated natural killer cells, and (3) monocytes vs. monocytes after 1 day in vitro macrophage differentiation'. As dataset (4), we combined all datasets into one and compared all resting vs. all activated cells.

We performed differential gene expression analysis of the resting vs. the activated states. We then correlated these single gene results with the single gene results from our Cesarean section and islet autoantibody status analysis. For monocytes, we observed a significant association, but no significant association was observed in any of the other comparisons (see Figures A-D below).

Remarkably, at pathway level, we observed a significant correlation between activated monocytes and Cesarean section ($r = 0.44$, $p = 0.001$) and islet autoantibody status ($r = 0.58$, $p < 0.001$), between activated CD4+ T-cells and Cesarean section ($r = 0.34$, $p=0.055$) and islet autoantibody status ($r = 0.46$, $p = 0.027$), and the combination of all 3 cell types with Cesarean section ($r = 0.31$, $p = 0.059$) and islet autoantibody status ($r = 0.46$, $p = 0.004$) (Figures E-H below). This provides empirical evidence of our hypothesis of immune activation at pathway level. No significant association was found for activated natural killer cells and Cesarean section ($r = 0.19$, $p = 0.259$) or islet autoantibody status ($r = 0.12$, $p = 0.538$).

Figure A:

A: Correlation of single gene effects in Cesarean section compared to inactivated vs. activated CD4+ cells (r = -0.051, p = 0.184). **B:** Correlation of single gene effects in islet autoantibody status compared to the inactivated vs activated CD4+ cells (r = -0.000, p = 0.991).

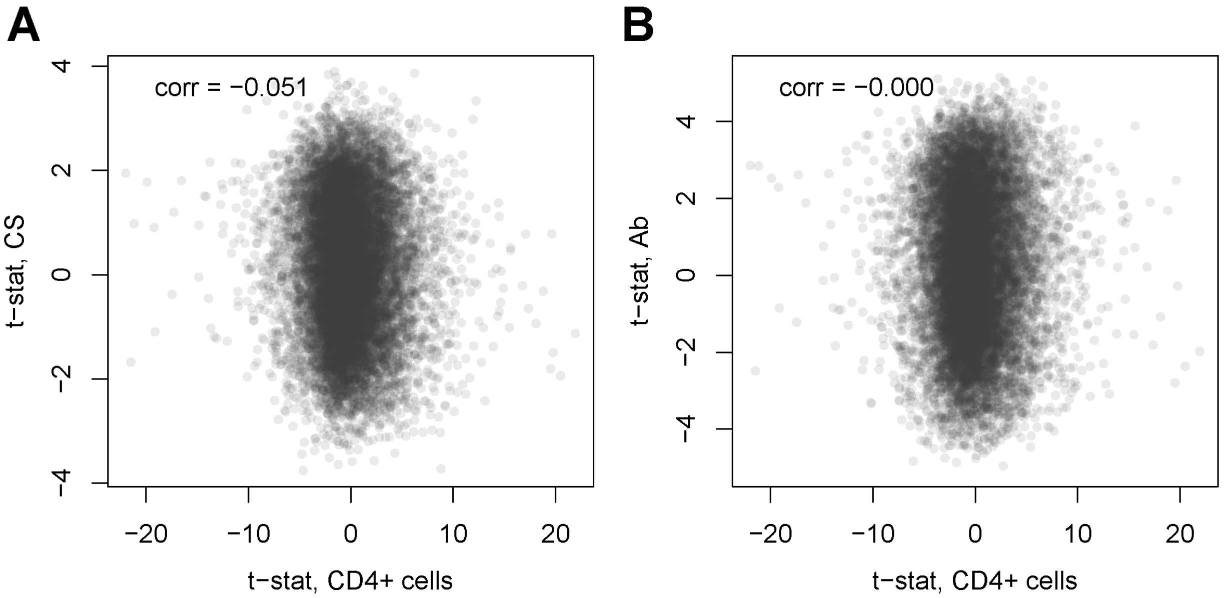

Figure B:

A: Correlation of single gene effects in Cesarean section compared to inactivated vs. activated monocytes (r = -0.184, p < 0.001). **B:** Correlation of single gene effects in islet autoantibody status compared to the inactivated vs activated monocytes (r = -0.188, p = 0.018).

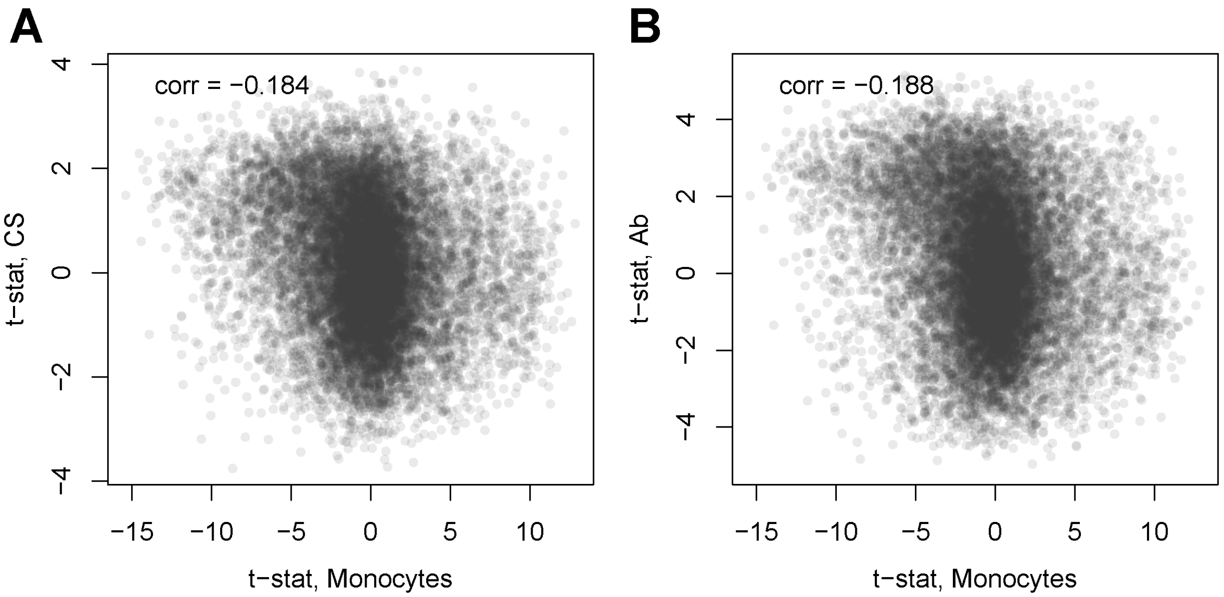

Figure C:

A: Correlation of single gene effects in Cesarean section compared to inactivated vs. activated natural killer cells (r = -0.043, p = 0.509). **B:** Correlation of single gene effects in islet autoantibody status compared to the inactivated vs activated natural killer cells (r = 0.007, p = 0.926).

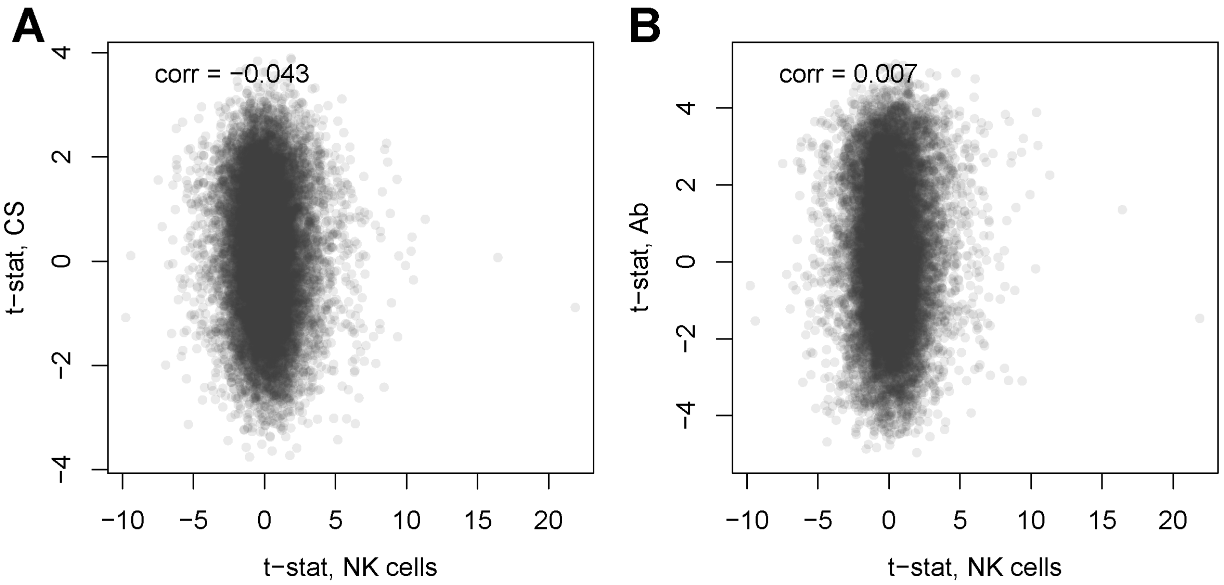

Figure D:

A: Correlation of single gene effects in Cesarean section compared to inactivated vs. activated CD4+ cells, monocytes, and natural killer cells (r = -0.172, p = 0.014). **B:** Correlation of single gene effects in islet autoantibody status compared to the inactivated vs activated CD4+ cells, monocytes, and natural killer cells (r = -0.133, p = 0.081).

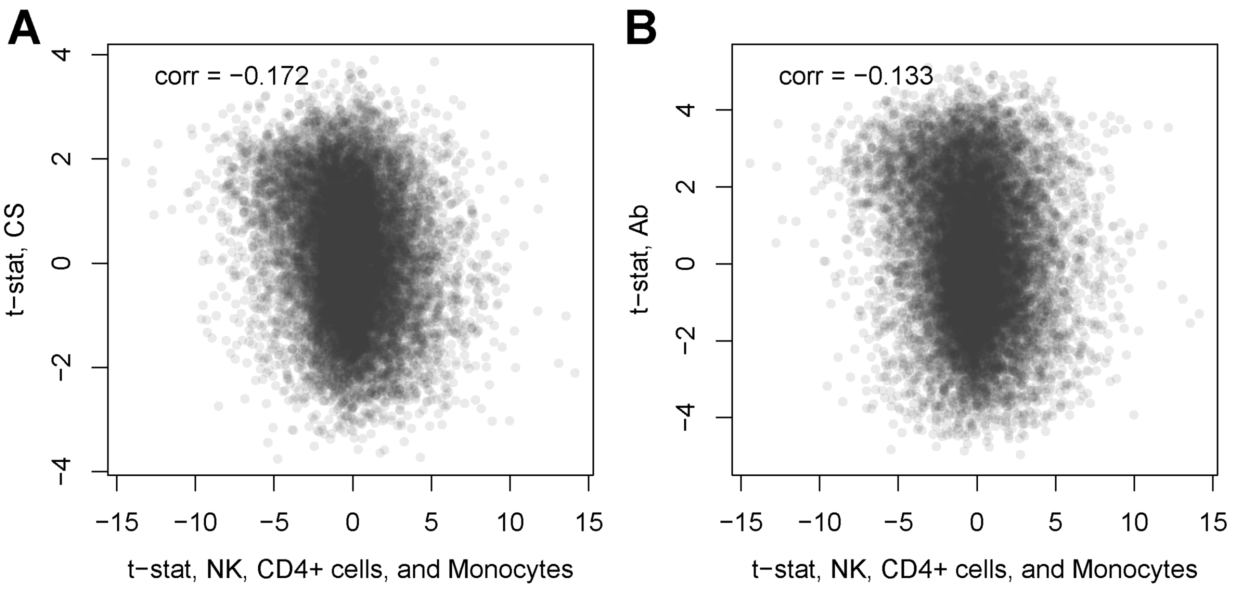

Figure E:

A: correlation of pathway associations in Cesarean section compared inactivated and activated pathways in CD4 T-cells (r = 0.339). **B:** Permutation-based assessment of correlation (p = 0.055). **C:** correlation of pathway associations in islet autoantibody status compared inactivated and activated pathways in CD4 T-cells (r = 0.455). **D:** Permutation-based assessment of correlation (p = 0.027).

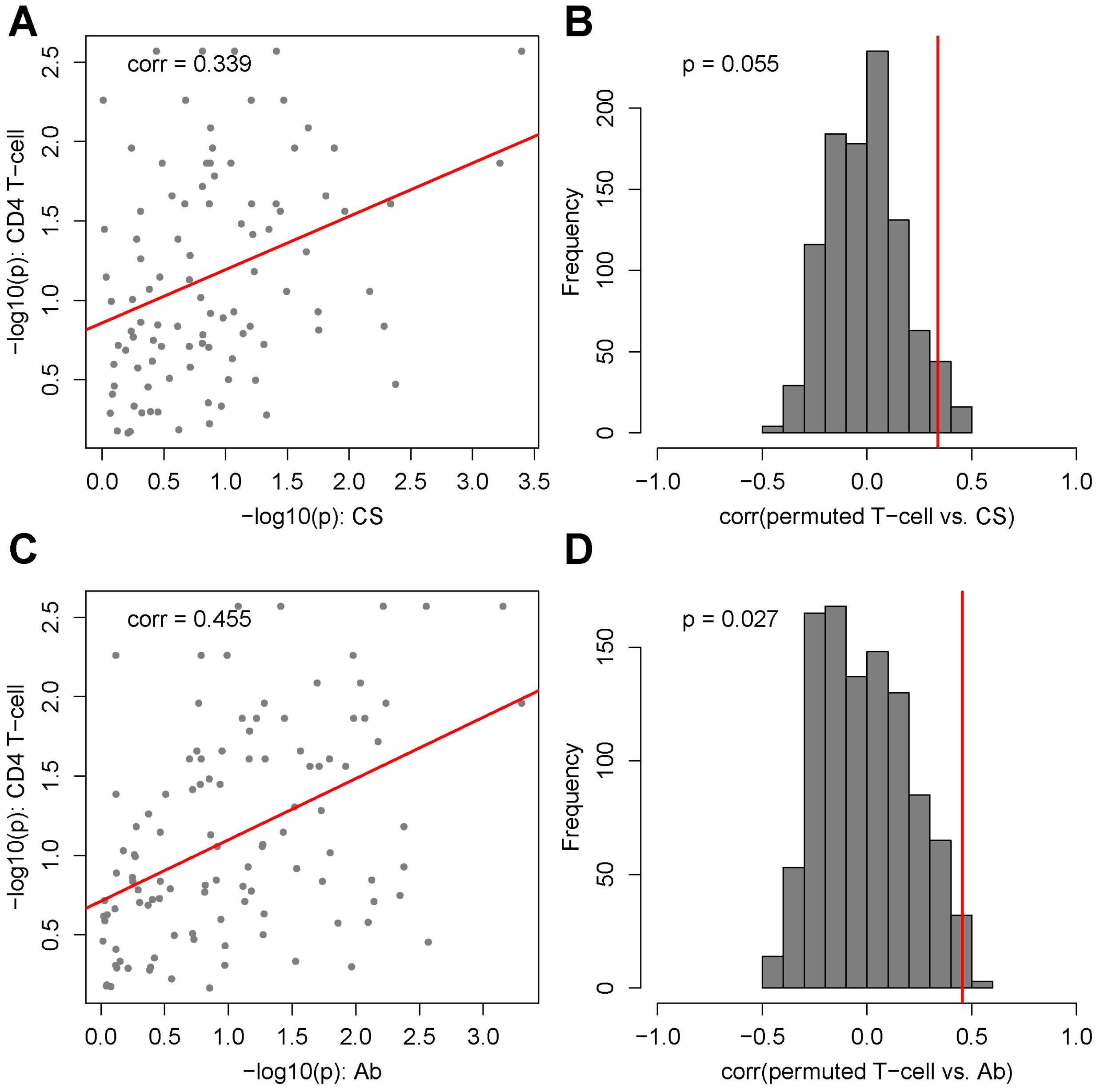

Figure F:

A: correlation of pathway associations in Cesarean section compared inactivated and activated pathways in monocytes (r = 0.441). **B:** Permutation-based assessment of correlation (p = 0.001). **C:** correlation of pathway associations in islet autoantibody status compared inactivated and activated pathways in monocytes (r = 0.580). **D:** Permutation-based assessment of correlation (p < 0.001).

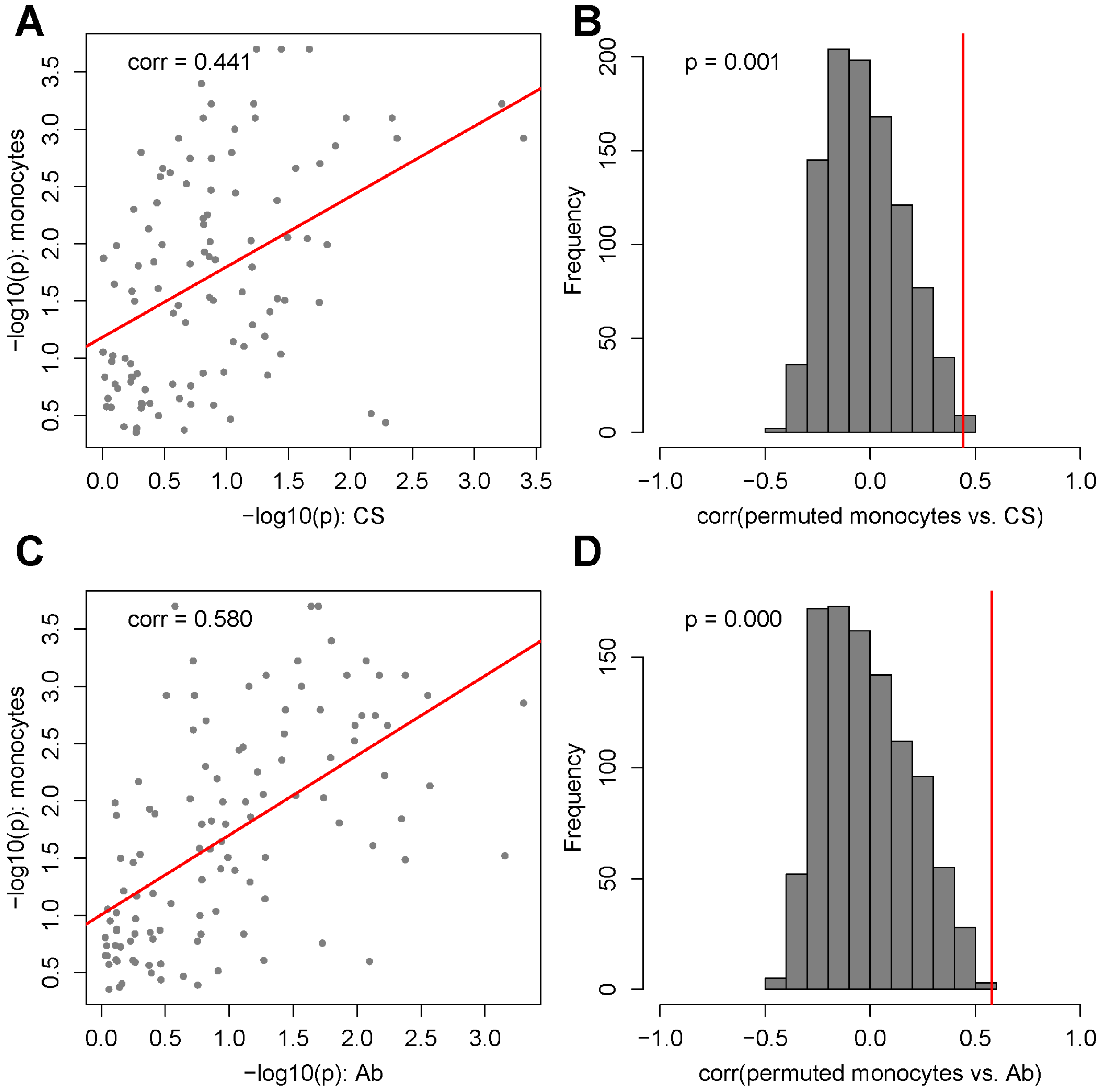

Figure G:

A: correlation of pathway associations in Cesarean section compared inactivated and activated pathways in natural killer cells (r = 0.186). **B:** Permutation-based assessment of correlation (p = 0.259). **C:** correlation of pathway associations in islet autoantibody status compared inactivated and activated pathways in natural killer cells (r = 0.115). **D:** Permutation-based assessment of correlation (p = 0.538).

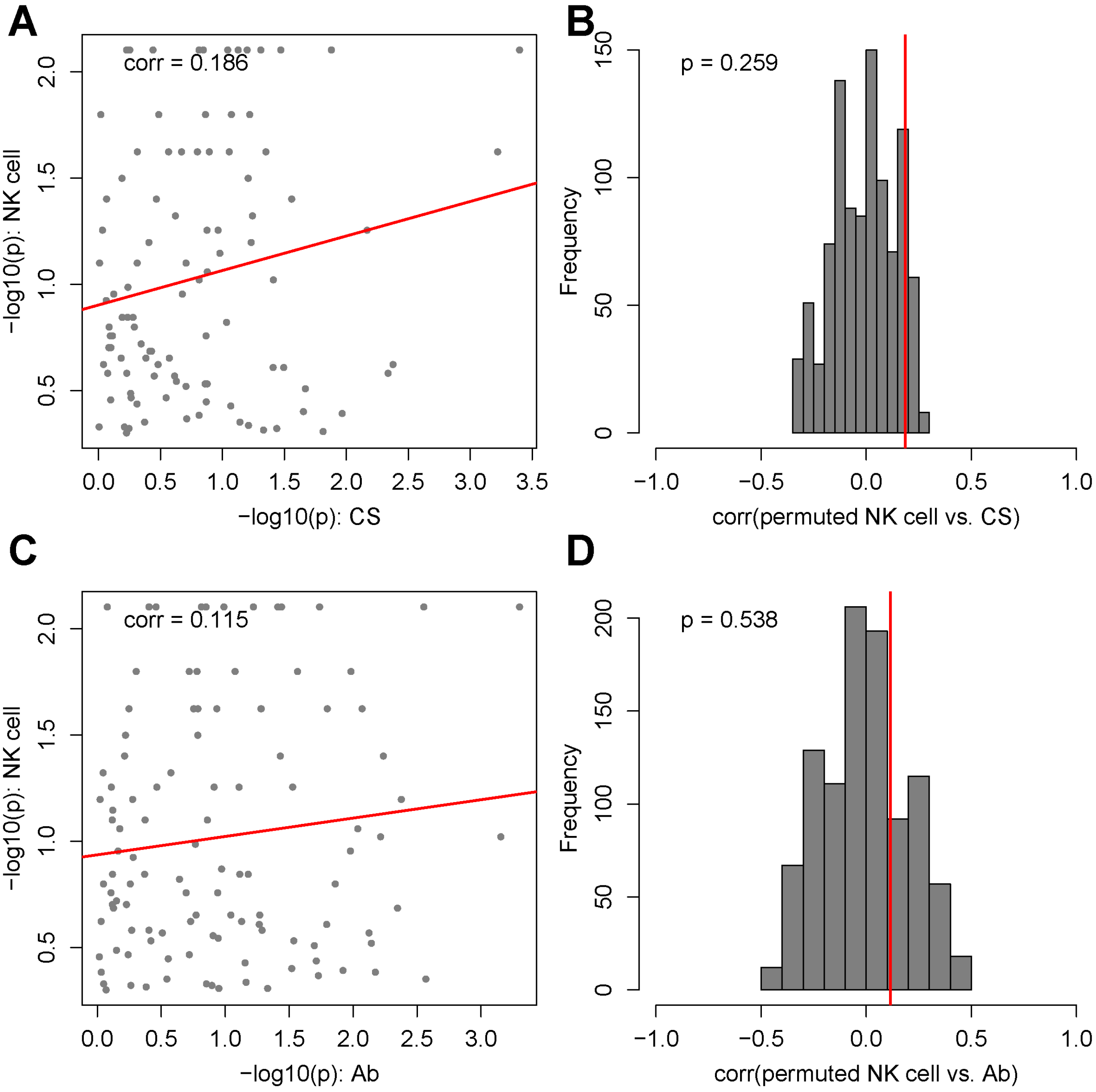

Figure H:

A: correlation of pathway associations in Cesarean section compared inactivated and activated pathways in a combination of CD4 T cells, monocytes, and natural killer cells (r = 0.305). **B:** Permutation-based assessment of correlation (p = 0.059). **C:** correlation of pathway associations in islet autoantibody status compared inactivated and activated pathways in a combination of CD4 T cells, monocytes, and natural killer cells (r = 0.462). **D:** Permutation-based assessment of correlation (p = 0.004).

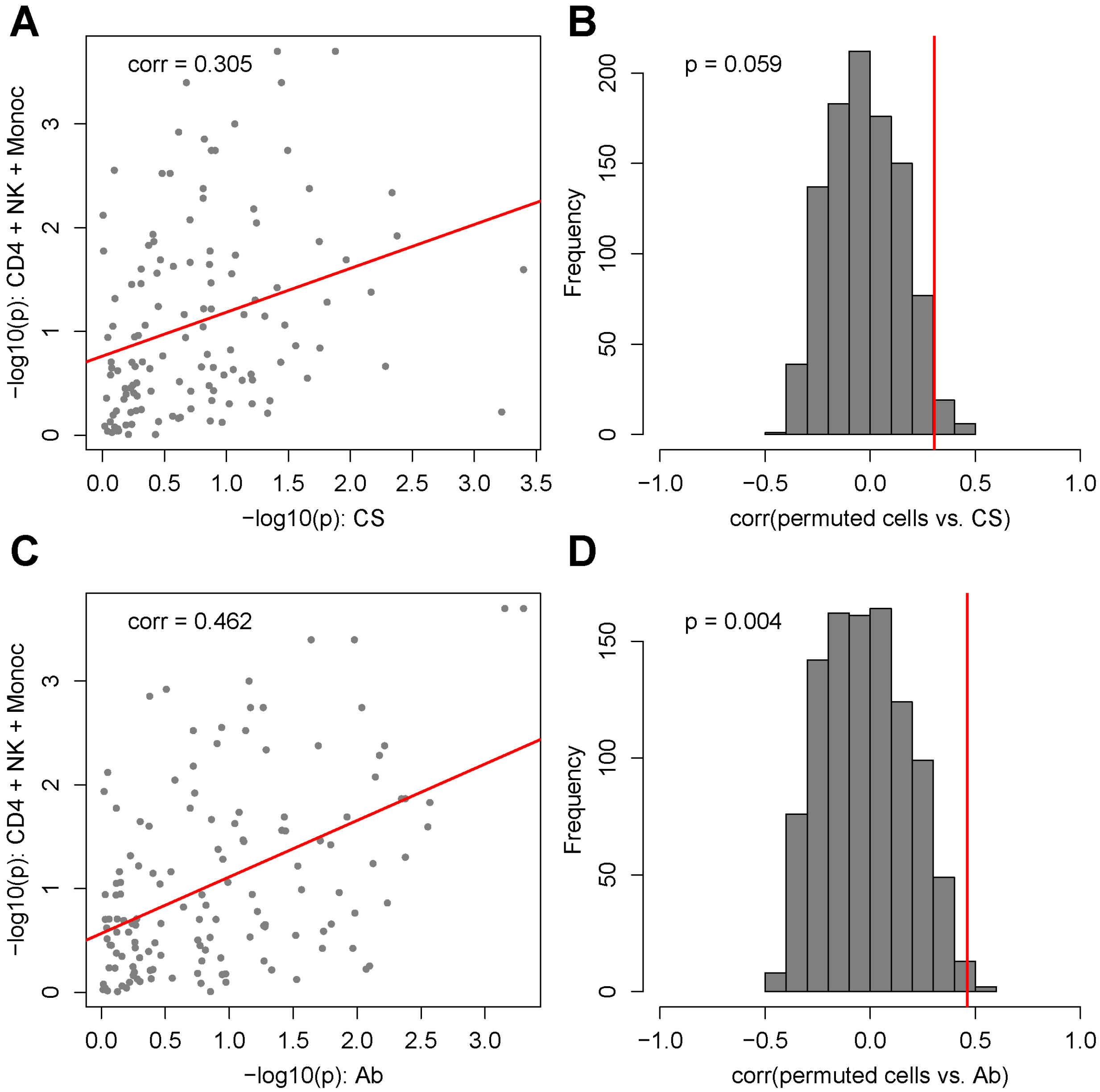

Supplement: Supplementary file 1 — Supplementary Information [file 41598_2019_42750_MOESM1_ESM.pdf]
